# Modeling of smoking intensity by age at smoking onset among Iranian adult male using generalized additive model

**DOI:** 10.1038/s41598-022-21194-4

**Published:** 2022-10-06

**Authors:** Zohreh Manoochehri, Javad Faradmal, Abbas Moghimbeigi

**Affiliations:** 1 Department of Biostatistics, Student Research Committee, Hamadan University of Medical Sciences, Hamadan, Iran; 2 Modeling of Noncommunicable Diseases Research Center, Department of Biostatistics, School of Public Health, Hamadan University of Medical Sciences, Hamadan, Iran; 3 Department of Biostatistics and Epidemiology, Faculty of Health & Health, Safety and Environment Research Center, Alborz University of Medical Sciences, Karaj, Iran

**Keywords:** Health care, Medical research, Risk factors

## Abstract

Because the age at which a person first starts smoking has such a strong correlation with future smoking behaviours, it's crucial to examine its relationship with smoking intensity. However, it is still challenging to accurately prove this relationship due to limitations in the methodology of the performed studies. Therefore the main purpose of this study is to evaluate the potential risk factors affecting the intensity of smoking, especially the age of smoking onset among Iranian adult male smokers over 18 years of age using a generalized additive model (GAM). In GAM a latent variable with logistic distribution and identity link function was considered. Data from 913 Iranian male current smokers over the age of 18 was evaluated from a national cross-sectional survey of non-communicable disease (NCD) risk factors in 2016. Individuals were classified into: light, moderate, and heavy smokers. A GAM was used to assess the relationship. The results showed that 246 (26.9%) subjects were light smokers, 190 (20.8%) subjects were moderate smokers and 477 (52.2%) subjects were heavy smokers. According to the GAM results, the relationship was nonlinear and smokers who started smoking at a younger age were more likely to become heavy smokers. The factors of unemployment (OR = 1.364, 95% CI 0.725–2.563), retirement (OR = 1.217, 95% CI 0.667–2.223), and exposure to secondhand smoke at home (OR = 1.364, 95% CI 1.055–1.763) increased the risk of heavy smoking. but, smokers with high-income (OR = 0.742, 95% CI 0.552–0.998) had a low tendency to heavy smoking. GAM identified the nonlinear relationship between the age of onset of smoking and smoking intensity. Tobacco control programs should be focused on young and adolescent groups and poorer socio-economic communities.

## Introduction

Smoking is a social epidemic^1^ that, despite its adverse effects on health and the economy, continues to be one of the top causes of preventable disease and death globally^2^. Tobacco use accounts for 15.4% of all deaths worldwide in 2019^2^. Smoking is one of the main factors in causing and aggravating various diseases such as chronic obstructive pulmonary disease, neurological diseases ,cardiovascular diseases^3^, and various cancers^4^. It is estimated that 1.2 million deaths per year worldwide are due to secondhand smoking (SHS), most of which occur in children under 10 years of age^5^. According to the latest reports in 2016, the prevalence of daily smoking in Iran is 9.7% and it is significantly higher among men (19.6%) than women (0.9%)^6^. This is because men could be more prone to turn to smoking as a result of a stronger predisposition to risky behaviours and dealing with numerous job-related difficulties, family and social duties.

Smokers can be classified into three categories based on their smoking intensity: light, moderate, and heavy^7^. Based on the results of previous studies, there are important differences between these three groups. For example, heavy smokers are more exposed to the negative effects of smoking such as low quality of life and it is also very difficult for them to quit smoking^8^. In addition, data from large population studies show that light smokers are 2 to 5 times more likely to experience respiratory symptoms and heart disease compared with nonsmokers^9^. Identifying the factors influencing the intensity of smoking such as socio-demographic differences, type of smoking habit, age at smoking onset, and ability to quit smoking can provide the information needed to adopt and implement tobacco control policies^1^. The age at smoking onset can significantly predict future smoking patterns and related health consequences^10^. According to a study by Nash et al., the age at smoking onset was strongly associated with death after the age of 70, so that current smokers who started smoking at a younger age were at higher risk for death compared to smokers who started later^11^. Based on a considerable body of studies^12,13^, it is commonly believed that the early age at smoking onset predicts heavy smoking in the future. Relevant researched, on the other hand, have either not explicitly tested this association or have been hampered by methodological flaws^14^. Many studies, for example, have used binary or grouped variables to display age at onset^12,13,15^, resulting in the loss of potentially valuable information about the onset path over time and the inability to assess a specific period of life, such as adolescence, when people are particularly vulnerable^14^. Also we can point to the lack of an appropriate model to detect this relationship.

Usually to describe the relationship between risk factors and outcome, classical statistical models such as the linear regression model are used. In reality, however, we frequently need to model more complex phenomena than those depicted by linear relationships. Among these, generalized additive models (GAMs) can be considered as an intermediate between classical models and machine learning models which can both fit complex and nonlinear relationships and act very strongly in terms of interpreting and understanding the fitted model. In fact, GAMs allow us to model nonlinear relationships along with linear relationships with high flexibility^16^.

In the present study, there is also a nonlinear and unknown relationship between one or more features such as age at smoking onset and outcome under consideration (smoking intensity) that conventional statistical models will not be able to identify this type of relationship. Therefore, due to the relatively high prevalence of smoking among Iranian men^6^ and its harmful role in causing various diseases, we try to use GAMs in order to evaluate the factors affecting the intensity of smoking, especially the age of smoking onset among Iranian adult male smokers over 18 years of age. The findings of this study can be shared with health policymakers so that they can plan and implement initiatives to reduce smoking intensity by focusing on these factors.

## Materials and methods

### Study setting, population, and sampling method

In this cross-sectional study (approved by “The Ethics Committee of the Hamadan University of Medical Sciences”; NO. IR.UMSHA.REC.1399.105 ) we analysed data related to tobacco use in a national cross-sectional study entitled “survey of risk factors for non-communicable diseases (NCDs) in 2016” conducted by the NCDs research center of Iran in order to assess the relationship between the age at which people start smoking and the intensity of smoking. Under the direction of the World Health Organization, a survey of risk factors for NCDs is conducted in the form of a study of the care system for risk factors for NCDs (WHO). Its overarching purpose is to build the infrastructure needed for global NCD risk factor management, with a focus on developing countries, as well as to provide global sources of information on the process and distribution of risk factors.

The study target group was adults over 18 years old and sampling was done from all provinces of Iran except Qom province. Samples were selected using multi-stage cluster sampling method.

### Data collection

The WHO STEPwise method for risk factor Surveillance, is called STEPs^17^.To acquire data on tobacco, researchers utilised the WHO's standard STEPs study questionnaire, which was self-reported. For this purpose, the questionnaire was translated from English to Persian by two experts and was again translated from Persian to English by two other experts so that the translation expresses the intended goals. In order to assess the validity of the questionnaire and its questions, the opinions of experts in the field were used. Cronbach's alpha coefficient was utilised to evaluate the questionnaire's reliability, and the determined value was 80 percent. The study protocol contains information about this survey^17^. All methods were performed in accordance with the relevant guidelines. After applying the exclusion criterion, which will be discussed in the next section, 913 people were studied in this study. In this cross-sectional study, 98.9% of participants gave full answers to the smoking status questionnaire.

### Predictor and outcome variables

Predictive variables were considered in terms of features related to demographic variables, economic status, and smoking behavior. Demographic variables included age, residence (urban/rural), marital status (married/other: single, divorced, widowed), level of education (Illiterate, lower than diploma/diploma and higher). variables including health basic insurance status (yes/no), monthly household income level (more than $ 175 vs $ 175 or less: based on the basic salary of the Ministry of Labor of Iran) and employment status (employee/worker/self-employed/retired/unemployed/others including: student, soldier, unpaid work), were considered as economic predictors. Based on definition the Merriam-Webster Dictionary, a worker is “a person who does a particular job to earn money.” Whereas, an employee is “a person who works for another person or for a company for wages or a salary.”^18^.

Some questions were asked to the participants in order to assess smoking behaviour among the Iranian population. Iranian Participants were divided into three categories: never smoker, former smoker, and current smoker, based on their answers to the questions "Have you ever smoked" and "Do you smoke now?". Non-smokers were participants who answered "no" to both questions. If participants answered "yes" to the first question and "no" to the second question, they would be classified as former smokers. Participants who answered "yes" to both of the above questions were considered current smokers. In the present study, non-smokers as well as former smokers who quit, that is, people whose answers to the question "Have you quit daily smoking?" were "yes" excluded from the study, and only the current smokers were studied. By applying this exclusion criterion, the sample size was reduced to 913 people. As mentioned in the introduction, one of the features that its relationship to the outcome (smoking intensity) is challenging, is the age at smoking onset. This feature was measured using the question “at what age did you start smoking?” Another aspect that is an essential indication of nicotine dependency, in addition to the age of smoking initiation, is trying to quit smoking^19^, which we tested using the question "Have you tried to quit smoking in the previous 12 months?" Because smokers are the most vulnerable group to smoking-related health risks^20^, the question "Have your doctor or healthcare professional advised you to quit smoking in the last 12 months?" also considered as a possible predictor of outcome. We assessed exposure to secondhand cigarette smoke using the question "Has anyone in your house or workplace smoked in your presence in the last 30 days?" in addition to the characteristics described in regard to smoking behaviour.

The study's outcome is that the intensity of smoking was examined using the question "How many cigarettes do you currently smoke each day?" The answer to this question was classified into three categories: less than 10 cigarettes/day as a light smoker, 10–19 cigarettes/day as moderate, and larger or equal to 20 cigarettes/day as a heavy smoker^7^.

### Statistical methods and software

After deleting the missing data, we described the sample using appropriate descriptive statistics. Then we used one-way analysis of variance to compare the mean age and age at smoking onset in three groups of smokers and to assess the association between categorical/discrete variables and response variables, the chi-square test was used. After performing univariate analysis, we used GAM regression to adjust the potential confounder effect by each of the explanatory variables. As potential interactions in the GAM, we entered the interactions between the variables of the level of education and employment status, as well as the interaction between the variables of employment status and exposure to secondhand smoke at work.We entered the variables into the model as follows: based on the literature review, the factors that had the largest impact on the outcome (marriage status^21^, level of education, residence^22^, employment status^20,23^, and monthly household income level^22^) were kept fixed in the model and additional variables were selected using the backward method. After selecting the variable by the mentioned method, the effect of each feature on the outcome is expressed using the odds ratio (OR) criterion. Also, nonlinear relationships between age at smoking onset as well as age with smoking intensity were presented graphically.

### Generalized additive models (GAMs)

GAM^24^ is an extension of the generalized linear model that is not sensitive to the assumption that the relationship between the covariates and the expected value of the response variable is linear. The general structure of GAM can be presented as follows:

1 $$g\left(E\left(y\right)\right)={X}_{i}\theta +\sum_{j=1}^{p}{f}_{j}({x}_{ji})$$

where g is the link function, y is the response variable, θ is the vector of the fixed parameters, ${X}_{i}$ is a row of the design matrix, and ${f}_{j}s$ are smooth functions of the covariates ${x}_{k}$. The normal, gamma, binomial, and Poisson distributions, which use identical, inverse, logit, and log link functions, respectively, are the most prevalent of the distributions accessible in GAM for the response variable. In GAM, the main issue is estimating the unknown function f(.). This unknown function, which describes the relationship between the explanatory variables and the response variable, is estimated using the data itself and a variety of smoothing methods^25^. Smoothing is the process of fitting a derivative curve to data. Smoothing can be accomplished in a variety of ways, with splines being one of the most common and powerful. A spline is a curve made up of polynomial sections that are uniformly connected at points called nodes^16^. Thin plate regression splines, cubic regression splines, and P-splines are the most prevalent splines. The effective degree of freedom is used to calculate the degree of curvature of a smooth curve in splines (edf). If edf = 1 then the estimated relationship will be linear. A larger edf would indicate a more complex relationship between the explanatory variable and the response variable^26^. In GAMs, nonparametric terms are represented using penalized spline regression with smoothing parameters selected using one of the criteria GCV/UBRE/AIC/REML or by regression splines with fixed degrees of freedom^27^. Refer to reference^24^ for more information on this topic and other estimation methods.

### Software

SPSS software version 24 was used to describe the data. To fit the GAM, we used the ocat function included in the mgcv package in R4.0.3 software. The expected value of a latent variable that follows the logistic distribution is estimated using a linear predictor with the identity link function in this package for the ordinal categorical data that the current study is based on. The probability of belonging to each category of the ordinal categorical variable is determined by the probability of finding this latent variable between specific cut-points^27^.

### Ethical approval and consent to participate

The study was approved by research ethics committee of Hamadan University of Medical Sciences. The written informed consent was obtained from all the participants.

## Results

Among the included 913 male subjects, 246 (26.9%) subjects were light smokers, 190 (20.8%) subjects were moderate smokers and 477 (52.2%) subjects were heavy smokers. The mean (standard deviation) age of all participants was 47.38 (13.48) years. Table 1 shows some of the demographic features of the study participants in terms of low, medium, and heavy smoking. According to the results of this table, the mean age of heavy smokers was higher than the two groups with light and moderate consumption (P-value = 0.008). While the mean age at smoking onset in the heavy smokers group was lower than the other two groups (P-value < 0.001). Although a higher proportion of participants (53.1%) had income levels over $175, most heavy smokers (50.5%) had lower income levels than light and moderate smokers (P-value = 0.048). Among all participating smokers in the study, 75.2% had made no try to quit smoking in the past 12 months, and this percentage was higher for heavy smokers (79.9%) compared to the other two groups (P-value = 0.001).

**Table 1:** Demographical feature and status.

Quantitative features	Light smokers (Mean ± SD)	Moderate smokers (Mean ± SD)	Heavy smokers (Mean ± SD)	Test Statistic	p-value	
Age (year)	45.71 ± 14.04)	46.24 ± 13.69	48.69 ± 12.99	F = 4.86	0.008*	
Age at smoking onset (year)	23.39 ± 8.97	21.87 ± 8.25	20.07 ± 6.63	F = 15.86	< 0.001*	

Qualitative features		Count (%)				p-value
		Light smokers (n = 246)	Moderate smokers (n = 190)	Heavy smokers (n = 477)	Total	
Residence	Urban	167(67.9)	122(64.2)	299(62.7)	588(64.4)	0.383
	Rural	79(32.1)	68(35.8)	178(37.3)	325(35.6)	
Marital status	Married	224(91.1)	165(86.8)	431(90.4)	820(89.8)	0.301
	Other	22(8.9)	25(13.2)	46(9.6)	93(10.2)	
Employment Status	Employee	25(10.2)	19(10.0)	36(7.5)	80(8.8)	0.265
	Worker	37(15.0)	26(13.7)	61(12.8)	124(13.6)	
	Self-employed	137(55.7)	92(48.4)	258(54.1)	487(53.3)	
	Retired	26(10.6)	24(12.6)	58(12.2)	108(11.8)	
	Unemployed	19(7.7)	22(11.6)	57(11.9)	98(10.7)	
	Other	2(0.8)	7(3.7)	7(1.5)	16(1.8)	
Level of education	Illiterate	24(9.8)	20(10.5)	68(14.3)	112(12.3)	0.094
	Lower than diploma	157(63.8)	120(63.2)	315(66.0)	592(64.8)	
	Diploma and higher	65(26.4)	50(26.3)	94(19.7)	209(22.9)	
Income level	$ 175 or less	101(41.1)	86(45.3)	241(50.5)	428(46.9)	0.048*
	more than $ 175	145(58.9)	104(54.7)	236(49.5)	485(53.1)	
Basic insurance	No	25(10.2)	16(8.4)	55(11.5)	96(10.5)	0.487
	Yes	221(89.8)	174(91.6)	422(88.5)	817(89.5)	
Trying to quit	No	165(67.1)	141(74.2)	381(79.9)	687(75.2)	0.001*
	Yes	81(32.9)	49(25.8)	96(20.1)	226(24.8)	
Physician recommendation	No	154(62.6)	104(54.7)	271(56.8)	529(57.9)	0.198
	Yes	92(37.4)	86(45.3)	206(43.2)	384(42.1)	
Expose to secondhand smoke at home	No	133(54.1)	101(53.2)	222(46.5)	456(49.9)	0.097
	Yes	113(45.9)	89(46.8)	255(53.5)	457(50.1)	
Expose to secondhand smoke at work	No	149(60.6)	105(55.3)	289(60.6)	543(59.5)	0.414
	Yes	97(39.4)	85(44.7)	188(39.4)	370(40.5)	

*Significant test in level 0.05.

In order to assess the effect of the age at smoking onset on the intensity of smoking by adjusting the effect of other features under consideration, we fitted the GAM. The result of GAM is reported in Table 2 in both parametric and non-parametric parts. In the parametric part of the GAM, the estimation of the coefficients of variables, their significance, and also the corresponding OR (OR of heavy smokers vs light and moderate smokers) are reported. Income level and try to quit smoking features significantly predicted the intensity of smoking. None of the interactions between the pair of features of the level of education and employment status, as well as the interaction between the features of employment status and exposure to secondhand cigarette smoke at the workplace, were significant. The parametric section of GAM showed that the odds of more heavy smoking (heavy vs moderate and light) among smokers with lower than diploma and diploma or higher were 0.932 and 0.809 times less than illiterate smokers. To put it another way, a higher education level was a protective factor for higher consumption. Single subjects had a higher risk of smoking more intensely than married subjects (OR = 1.409). Furthermore, those who were exposed to secondhand smoke at home had 1.364 times the probability of consuming more intensely than non-exposed smokers. According to the results, the risk of more intense smoking approximately was the same among urban and rural areas (OR = 0.916). Compared to employees, the risk of more intense smoking was higher among the unemployed (OR = 1.364), retirees (OR = 1.217), self-employed (OR = 1.192), and workers (OR = 1.182), respectively. In addition, high-income smokers have less tendency to intense smoking than low-income smokers (OR = 0.742). Also, trying to quit for the past 12 months was not associated with heavy smoking (OR = 0.629).

**Table 2:** Association of intensity of cigarette smoking (heavy vs light and moderate smokers) and independent variables measured by the generalized additive model.

Estimation of parametric coefficients				
Variable	Estimate (SE)	P-value	OR	95% CI
**Marital status (married as reference)**				
Other	0.343 (0.237)	0.148	1.409	0.885–2.242
**Level of education (illiterate as reference)**				
Lower than diploma	−0.069 (0.235)	0.767	0.932	0.589–1.479
Diploma and higher	−0.212 (0.272)	0.437	0.809	0.475–1.379
**Residence (rural as reference)**				
Urban	−0.088 (0.149)	0.556	0.916	0.684–1.223
**Employment status (employee as reference)**				
Worker	0.167 (0.287)	0.561	1.182	0.673–2.074
Self-employed	0.176 (0.242)	0.468	1.192	0.742–1.916
Retired	0.197 (0.307)	0.522	1.217	0.667–2.223
Unemployed	0.310 (0.322)	0.336	1.364	0.725–2.563
Other	0.178 (0.513)	0.728	1.195	0.437–3.266
**Income level ($ 175 or less as reference)**				
More than $ 175	−0.298 (0.151)	0.048*	0.742	0.552–0.998
**Try to quit (No as reference)**				
Yes	−0.463 (0.150)	0.002*	0.629	0.469–0.844
**Expose to smoke at home (no as reference)**				
Yes	0.310 (0.131)	0.018*	1.364	1.055–1.763

**Approximate significance of smooth terms (Non-parametric)**				
**Smooth covariates**	**edf**	**Ref.df**	**Chi.sq**	**P-value**
Age (year)	2.974	9	25.26	< 0.001*
Age at smoking onset (year)	1.913	9	41.41	< 0.001*

*Significant at 0.05.

In the non-parametric part, which is presented in the second part of Table 2, the reported edf values for the variables of age at smoking onset and age are 1.913 and 2.974, respectively. Since these numbers are greater than one (approximately from degree two for age at smoking onset and three for age) is indicative of a nonlinear relationship with the outcome variable (intensity of smoking) so these nonlinear relationships are also statistically significant (P-value < 0.001).

**Figure 1:**
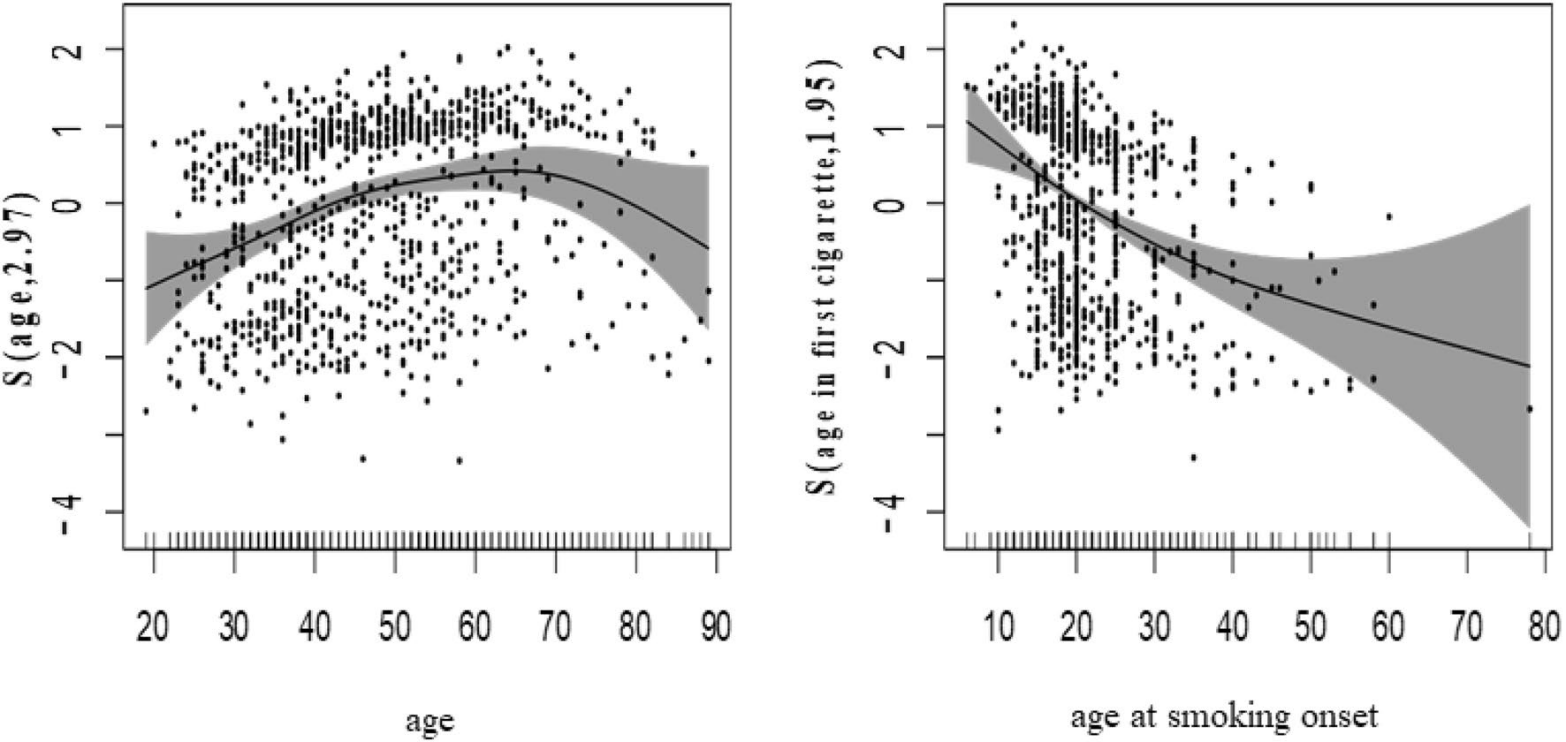
Estimating the smooth function of the relationship between: (left) age and smoking intensity, (right) age at smoking onset and smoking intensity. The numbers displayed in brackets in the y-axis title represent the edf of smooth curves. The linear predictor scale is used to present the results. The ‘rug plot’ at the bottom of each graph indicates the covariate values. The points on the graph are residuals. The grey region represents the Bayesian confidence interval of 95%.

The plots of predicted smooth function with 95% Bayesian confidence interval of these two factors in Fig. 1 also shows the nonlinear relationship of these two factors with smoking intensity as an outcome.

**Figure 2:**
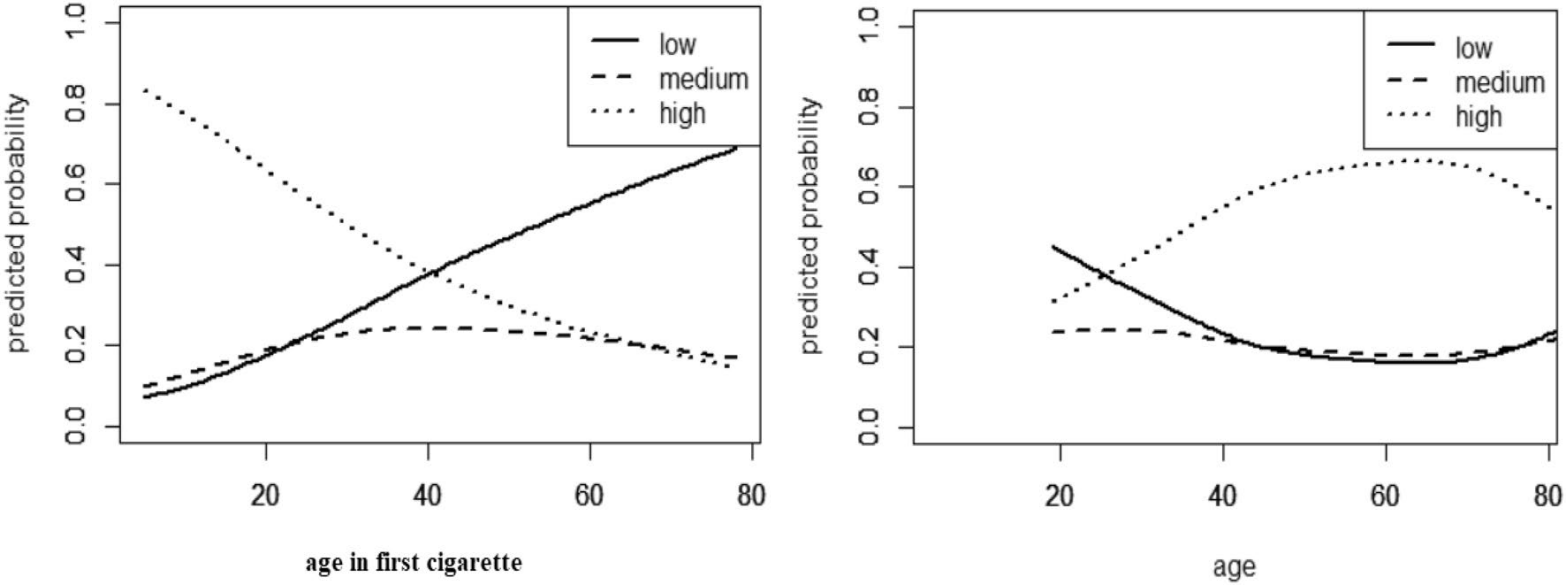
Plot of response probabilities in three groups with low, moderate, and heavy consumption vs. age at smoking onset (left) and age (right). The sum of the three probabilities is equal to one.

Figure 1 only shows the functional form of the relationship between the features and the outcome under consideration (smoking intensity). In order to determine the effect of the variable of age at smoking onset on the estimated probabilities of the three outcome groups, Fig. 2 is presented. Based on the results of this figure (left panel), subjects who start smoking at a younger age are more likely to become heavy smokers. Conversely, subjects who start smoking at an older age are more likely to become light smokers than moderate or heavy-smokers. Also, according to Fig. 2 (right panel), subjects between the ages of 40 and 70 with more probability, smoke more daily cigarettes.

## Discussion

In this study, we assessed the factors affecting the intensity of smoking. One of the factors that its association with the intensity of smoking is important is the age at smoking onset variable. However, due to limitations in the methodology of the earlier conducted researches, correctly determining the connection of this feature with smoking intensity remains a challenge^14,28^. On the other hand, based on a review of the literature, no study has been conducted to assess the effect of the age at smoking onset on the intensity of smoking among Iranian adults. As a result, we used GAM as a flexible modelling tool to find nonlinear and complex associations between this variable and other features on smoking intensity in this study.

According to the results of this study, more than half of the smokers (52%) smoked more than 20 cigarettes a day. This result is inconsistent with the study of Okuyemi et al., because in this study, which was conducted on African American smokers, a significant proportion of smokers (about 40%) were light smokers^7^. Thus, given that smoking earlier makes people more addicted and smoking more cigarettes the prevention should begin at an early ages to prevent smoking related diseases and mortality. According to multivariate analysis, there was an inverse relationship between education levels and smoking intensity, implying that education levels had a protective impact, i.e., participants with lower levels of education were more likely to become heavy smokers. Rogers' idea, which was published in 1970, can explain this finding^29^. According to this notion, individuals and groups with more health benefits adopt new health ideas and practises sooner, while disadvantaged persons accept them later^30^.When compared to employees, the odds of more intense smoking were higher among the unemployed, retirees, self-employed, and workers. The positive association between retirement and unemployment with smoking is consistent with existing studies in the field of occupational status and health because, according to the findings of these studies, job loss increases unhealthy behaviors^23^. For example, in the Ayyagari study, it was reported that retirement increases the probability of intensity of smoking^23^. As mentioned, self-employed individuals had higher consumption intensity than employees. Managers smoke more than other jobs, according to the findings of a study by Wang et al.^20^. It's probable that, unlike employees, the high rate of smoking among managers and self-employed individuals can be attributed to superiors' lack of control over their actions. On the other hand, the lower smoking rate among employees can be justified by the fact that most organizations avoid employing smokers for reasons such as higher costs of health care, more absenteeism, and loss of productivity^31^. In the present study, most heavy smokers (50.5%) had lower income levels than light and moderate smokers. According to the study by Nketiah-Amponsah et al., rich Ghanaian males were less likely to smoke, while older men living in poorer areas were more likely to smoke^22^. Household income, on the other hand, was not a substantial predictor of smoking in a study by Villanti et al.^32^.This discrepancy could be attributable to differences in the target communities under investigation. For example, in addition to men, the study's target population included a subgroup of women who, on average, have lower income levels than men. According to the GAM, exposure to secondhand smoke at home had a strong relationship with smoking intensity, which is consistent with the results of the study by Itanyi et al.^33^.

Because most heavy smokers began smoking near the end of adolescence or in early youth in the current study, the mean age at smoking onset in the heavy smokers group (20.07 ± 6.63) was lower than in the other two groups. This is because the transition from adolescence to young adulthood is a vital period of life during which young people graduate from high school and leave home to attend college or find a suitable job. These changes typically lead to reduced parental control and changes in social networks and increase the vulnerability of this age group to substance use, including smoking^34^. Although it is believed that smoking begins in adolescence, studies have shown that the onset of smoking also occurs in later life. According to the results of the present study, only 9 subjects, less than 1% of the participants started smoking at the age of over 50 years. This means that if a person has not started smoking in adolescence and early adulthood, very unlikely to start smoking later in life. The people who begin smoking at a younger age are more dependent on nicotine and their consumption will increase, whereas those who begin at an older age will have less intensity of consumption (Fig. 2 left panel). This result is consistent with the study of Hamzeh et al.^1^. In this study, using the GAM model, in addition to age at smoking onset, we also assessed the nonlinear effect of age on smoking intensity (Fig. 2, right panel). As a result, people between the ages of 40 and 70 are more likely to smoke more cigarettes on a regular basis. According to a Ghanaian study, older males are more likely to smoke than their younger counterparts, and they also consume more^22^. Perhaps the probability of more smoking in this age group can be attributed to their younger years. Most likely, these individuals began smoking throughout their adolescence and youth, and their consumption has gradually escalated to the point where smoking has become a habit for them and quitting is quite tough in this age range^22^. In addition, adult men often have so many financial responsibilities so that some of them are unable to meet the basic needs of their lives and this has caused a lot of stress and anxiety therefore, these people turn to heavy smoking to escape the created anxiety and stress^35^. Since 12% of deaths worldwide are attributed to smoking among adults over 30 yaers comprehensive measures are needed to prevent nonsmokers from starting to smoke in order to reduce the burden of death caused by smoking^11^.

## Limitations

Because most epidemiological studies use self-reported data without any biomedical markers for smoking, one of the limitations of this type of study is exposure to potential biases such as recall bias^36^, from which our study also was no exception. Another limitation of this study was that although the samples used are representative of the data of the entire country, they do not include the female population due to the low response rate. It is suggested that in future studies, the age at smoking onset and other factors in women should also be investigated and compared with the male population.

## Conclusions

According to the results of this study, poorer socioeconomic status as well as starting smoking at a younger age is associated with heavy smoking. Also, men in the age group of 40 to 70 years had higher consumption intensity than other age groups. Therefore, more intensive consumption and its adverse consequences in adulthood can be predicted by these factors.

## Data Availability

The datasets presented in this article are not readily available because: The authors need to grant permission of the National Institute of Health of Iran. Requests to access the datasets should be directed to Ali-Asghar Kolahi, a.kolahi@sbmu.ac.ir.
